# Youth Understanding of Healthy Eating and Obesity: A Focus Group Study

**DOI:** 10.1155/2013/670295

**Published:** 2013-07-17

**Authors:** Allison C. Sylvetsky, Monique Hennink, Dawn Comeau, Jean A. Welsh, Trisha Hardy, Linda Matzigkeit, Deanne W. Swan, Stephanie M. Walsh, Miriam B. Vos

**Affiliations:** ^1^Nutrition and Health Science Program, Graduate Division of Biological and Biomedical Sciences, Laney Graduate School, Emory University, Atlanta, GA 30322, USA; ^2^Section on Pediatric Diabetes and Metabolism, National Institute of Diabetes, Digestive, and Kidney Diseases, National Institutes of Health, Bethesda, MD 20892, USA; ^3^Department of Global Health, Rollins School of Public Health, Emory University, Atlanta, GA 30322, USA; ^4^Department of Behavioral Science and Health Education, Rollins School of Public Health, Emory University, Atlanta, GA 30322, USA; ^5^Department of Pediatrics, School of Medicine, Emory University, Atlanta, GA 30322, USA; ^6^Children's Healthcare of Atlanta, Atlanta, GA 30322, USA

## Abstract

*Introduction*. Given the high prevalence of childhood obesity in the United States, we aimed to investigate youth's understanding of obesity and to investigate gaps between their nutritional knowledge, dietary habits, and perceived susceptibility to obesity and its co-morbidities. *Methods*. A marketing firm contracted by Children's Healthcare of Atlanta facilitated a series of focus group discussions (FGD) to test potential concepts and sample ads for the development of an obesity awareness campaign. Data were collected in August and September of 2010 with both overweight and healthy weight 4th-5th grade and 7th-8th grade students. We conducted a secondary analysis of the qualitative FGD transcripts using inductive thematic coding to identify key themes related to youth reports of family eating habits (including food preparation, meal frequency, and eating environment), perceived facilitators and barriers of healthy diet, and knowledge about obesity and its complications. *Results*. Across focus group discussions, mixed attitudes about healthy eating, low perceived risk of being or becoming obese, and limited knowledge about the health consequences of obesity may contribute to the rising prevalence of obesity among youth in Georgia. Most youth were aware that obesity was a problem; yet most overweight youth felt that their weight was healthy and attributed overweight to genetics or slow metabolism. *Conclusions*. Our analysis suggests that urban youth in Georgia commonly recognize obesity as a problem, but there is less understanding of the link to lifestyle choices or the connection to future morbidities, suggesting a need for education to connect lifestyle behaviors to development of obesity.

## 1. Introduction

The prevalence of childhood obesity in the United States has risen dramatically over the last three decades [1] and is the highest in the Southeastern region of the country [2]. Overweight youth are at risk of being obese during adulthood [3] and are likely to experience obesity-related chronic illness [4]. The increase in obesity and its comorbidities among youth is multifactorial in cause, including increased access to foods high in fats, added sugars and calories [5], increased eating outside the home [6], larger portion sizes [7], and a sedentary lifestyle [8]. The diversity of these contributors to childhood obesity has made it difficult to design simple, achievable, public health solutions.

Studies have been conducted to identify strategies to combat obesity among youth; yet much remains to be understood. A recent qualitative study found that barriers to a healthy family lifestyle included cost of healthy food, time and practicality, family preferences, and difficulty in changing habits [9]. In a recent focus group study evaluating lifestyle perceptions among family members as young as 8 years of age, additional themes that emerged included making healthy activities fun, stage of youth development, and individual, family, and community involvement [10].

In order to understand why current obesity prevention strategies have largely failed and to inform the development of more successful future techniques to combat childhood obesity, it is imperative that the understanding of obesity among children be explored. While prior studies have assessed youths' perceptions of their own weight status and have explored their lifestyle behaviors, less emphasis has been placed on understanding youths' familiarity with the causes and consequences of obesity. The objective of this study was to explore youths' understanding of obesity as a problem and to investigate gaps between their nutritional knowledge, dietary habits, and perceived susceptibility to obesity and its comorbidities. 

## 2. Materials and Methods

This study comprises secondary analysis of qualitative data collected by an Atlanta-based marketing company contracted by Children's Healthcare of Atlanta (Atlanta, GA, USA) as part of their efforts to develop a public health campaign to reduce obesity amongst youth in the state of Georgia (USA). These data were not collected with the intention of being used for scientific purposes and were not analyzed scientifically by the marketing agency which carried out the primary data collection. However, the questions asked and the dialogue that emerged yielded discussion about obesity-related topics that were of scientific interest. Although the data were originally collected for marketing purposes, the conversations provide a unique window to examine youths' knowledge and understanding of the relationship between behaviors and obesity. 

Ten focus group discussions were conducted by the marketing company during August and September of 2010, in two regions of Georgia. As displayed in Table 1, participants were aged between 9 and 14 years and enrolled in either the 4th/5th grade (aged 9–11 years) or in the 7th/8th grade (aged 12–14 years). These age groups were selected because late childhood and early adolescence are key time periods where children begin to become independent from their parents and are able to evaluate and alter their dietary habits and attitudes [11]. Focus group discussions were limited to five children per group to enhance the children's comfort level and to account for the age and maturity level of the participants.

Discussion groups were stratified by age, gender, and weight status to construct homogenous groups of participants which encourages participants to feel comfortable in sharing their thoughts and in light of the potentially sensitive nature of discussing obesity. Both overweight and normal weight youth were included in the study in order to learn what all youth understood about obesity and the behaviors related to obesity and the potential marketing effectiveness of the proposed public health campaigns. The marketing company recruited youth through databases allowing for direct parent/caregiver contact and by networking with organizations serving children. Parents were randomly contacted and then screened for their child's eligibility. Participation was limited to one eligible child per household, and child weight status was determined by parent report of the child's height and weight. Of 52 youth recruited, 41 participated in the focus group discussions. The moderator was a slightly overweight female aged approximately 40 years old, with 15 years of experience moderating focus groups. Trained marketing company staff members obtained informed consent and assent from each parent/caregiver and each participant, respectively, before the focus group discussion began. Each focus group discussion was both audio- and video-taped with permission; only the audiorecordings were used in our secondary analysis. 

*Data Analysis*. In the original analysis conducted by the marketing company, only text relating to youths' understanding and opinions of the marketing concepts and campaigns were analyzed. Thus, we did not analyze this information in our secondary analysis but instead focused on the understanding of the causes and consequences of obesity amongst youth. For the purpose of this analysis, the term “understanding” was defined as comprehending or having a mental grasp on the concept, such as understanding that obesity can be caused by eating too much, whereas “recognition” was defined as merely being able to identify obesity. We used the word “perception” to refer to interpretation of obesity in relation to the youths' daily lives.

We analyzed data from both overweight and normal weight youth in order to explore potential differences in understanding based on weight status, and because we were interested in learning what all youth knew about obesity and its causes and consequences. Although the marketing company asked similar research questions, their primary analysis was used to inform the development of a public health campaign to reduce obesity in the state of Georgia. Our secondary data analysis of the same focus group transcripts aimed to uncover themes in the transcripts and to gain an understanding of the context underlying the textual data. Our secondary analysis of the deidentified focus group transcripts was approved by the Institutional Review Board at Children's Healthcare of Atlanta. The research analyst (AS) analyzed the written transcripts and, when necessary, contacted the marketing company for clarification related to the study procedure.

We used inductive thematic analysis to identify major themes in the textual data [12, 13] which emerged from the content of the focus group discussion transcripts. Two members of the research team (AS, an obesity researcher & DS, a behavioral science expert) read and reread the transcripts to familiarize themselves with the data and identify any patterns that emerged. Codes were developed to document patterns that emerged from the data which addressed the primary research question of whether youth demonstrated an understanding of obesity and its causes and consequences and if they perceived themselves as susceptible to the development of obesity. Codes were then compared to increase intercoder reliability and combined into overarching themes. Themes were then compared within and between the ten focus group discussions. For example, we noticed numerous comments that contrasted healthy and unhealthy foods (theme in codebook, 1.1) and found that youth could sometimes identify healthy foods such as broccoli (subtheme 1.1.1) while other foods were more ambiguous such granola bars and vitamin water (subtheme 1.1.2) and called these sub-themes “easily identifies healthy food (1.1.1), difficulty identifying healthy food (1.1.2).” An example of our coding scheme is further described in Table 2.

Participant's statements were often coded with multiple codes. The coding process was terminated when no new themes emerged from the transcripts during codebook development. We compared categories and codes between individuals to explore patterns that emerged in the data based on participant characteristics. Illustrative quotations demonstrating important themes which repeatedly emerged across focus groups and which enhanced our understanding of the data were extracted separately. 

## 3. Results

Three themes emerged consistently across the ten focus groups that were related to youths' perceptions of their own lifestyle and the causes and consequences of obesity. The gender, age, and weight status of focus group participants are shown in Table 1. 

*Theme Number 1: My Mom Wants Me to Eat Healthy Foods Like Broccoli but It Looks Nasty and Tastes Gross*. Youth did not have difficulty describing nutritious foods when asked “what does healthy eating make you think of?”, although female participants expressed more positive attitudes toward actually consuming healthy foods. All youth described salads, vegetables, and fruit as nutritious, whereas fast food, fried food, and candy were described as unhealthy. One participant described healthy eating as *“you eat fruits and vegetables and you do not just sit around and eat chocolate bars all day” (7th/8th grade, overweight, female).*

Most youth reported learning what foods were healthy and unhealthy from their parents or from classes in their school. For example, when describing her mother's influence on her diet, one female youth stated *“When I ask her for snack foods she says no because you need to start eating healthier food” (7/8th grade, overweight, female)*, implying that the mother views snack foods as unhealthy and might offer healthier alternatives. In another discussion group, youth mentioned that the food pyramid was a resource that youth learned how to use in order to determine which foods are healthy. Youth said that they had learned about the food pyramid in 5th, 6th, and 7th grades in several classes, such as science class, family consumer sciences, health class, and home economics. In explaining the overlap between classes where the food pyramid was covered, one participant recalled that *“Health classes is talking about the food pyramid, but our home-economics is just teaching us about food and stuff, and what's nutritious and not” (7th/8th grade, normal weight, male).*

Youth attitudes towards consuming healthy and unhealthy foods were mixed. Female participants were more likely to report enjoying healthy foods and also expressed a greater desire to positively change their diet. A few females in particular stated that they had requested that their parents purchase healthy foods. In contrast, several male participants associated healthy food with tasting bad. One boy commented that *“They eat stuff that tastes nasty just to lose the weight” (7th/8th grade, overweight, male). *There were no differences in opinion based on weight status among males, while overweight females often expressed desire to improve their diet to include more healthy foods. 

In addition to portraying more positive attitudes toward healthy diet than males, females expressed an understanding of the connection between food choices and body weight. One female participant explained that *“We used to do fast food and now my mom cooks more because it's like healthier” (4th/5th grade, overweight, female)* and also commented that *“I can get fat eating. I try to eat vegetables and stuff.”* In contrast, when asked what healthy food makes him think of, one male stated that *“it looks nasty…you know how some foods just have a nasty look and it's good for you but it's like…uh, I do not know”* (7th/8th grade, normal weight, male).

*Theme Number 2: Obesity Is a Problem but It Does not Apply to Me*. Most youth were familiar with the term “obese” and believed that obesity was a problem in Georgia. One person defined obese as a term *“for people who do not necessarily have a disease but that get too fat” (7th/8th grade, overweight, male). *When asked about the percentage of youth that were overweight or obese in Georgia, estimates varied greatly, ranging from 10% to 50%. However, many overweight or obese youth did not recognize that they were themselves overweight in the focus groups. For example, one overweight participant stated that “*I'm very aware of it because somebody in my school is overweight*,” not appearing to recognize that he himself was also overweight. Overweight girls were more likely to recognize that they were overweight than overweight boys. Overweight females often reported feeling self-conscious or being scrutinized by their classmates while eating. One girl expressed* “…everyone looked at me like, I wonder what she's going to do…They're watching me. They're vultures.” (7th/8th grade, overweight, female).*

About half of our participants believed that overweight and obese classmates recognized that they were overweight, but only some believed that they were trying to change. Normal weight youth more frequently commented that overweight classmates did not care or did not know how to change compared to overweight participants. Meanwhile, normal weight participants did not report practicing vigilance in their own eating habits to prevent weight gain. Across both normal weight and overweight youth, there was an overwhelming belief that their overweight peers would not recognize that they were overweight unless someone in their class had picked on them for being heavy. Only in response to being ridiculed, and not due to concerns about short- or long-term health, would their peers be motivated to change their body and to achieve a normal weight. When asked what health habits they would change, many overweight youth responded that they would focus on household chores or personal hygiene while lifestyle modifications and dietary improvements were rarely mentioned. In addition, when asked if overweight youth (in general) were doing anything to achieve a healthy weight, several participants responded that overweight youth do not care about their weight and “*will just eat whatever they want” and “not do anything about it*.”

*Theme Number 3: Everyone Is Made Differently and It Does not Matter If You Are Fat*. Youth demonstrated a limited understanding of the causes and consequences of obesity yet most youth, regardless of weight status, recognized that you can gain weight from eating. One girl said *“I do not like that [the appearance of her stomach] because it makes me feel like I'm getting fatter, so I'm trying to stop eating all the time” (4th/5th grade, overweight, female). *However, many overweight youth attributed overweight to fate, genetics, or metabolism, whereas normal weight youth commonly associated overweight with poor lifestyle choices. One overweight girl stated that *“everybody's made differently and God made us that way” (4th/5th grade, overweight, female). *Another overweight female commented “*Sometimes people have strong metabolisms or weak ones…like you bite something and you gain five pounds” (7th/8th grade, overweight, female).* Overweight participants often said that they did not like the word fat or the word obese, most often because it was hurtful and because being obese does not make you a bad person. Meanwhile, participants in the normal weight focus groups were more likely to provide causal explanations of obesity. For example, one normal weight participant stated “*if you do not eat healthy or exercise, then you can become obese and all of that stuff*” (7th/8th grade, normal weight, female). Another normal weight female elaborated on this comment in saying “*and then you cannot function as well, as someone who is not overweight*” (7th/8th grade, normal weight, female).

Normal weight individuals felt that resolving obesity is a long-term, multifaceted approach, requiring time, commitment, and support, while many overweight individuals expressed frustration and helplessness. For example, while most youth connected weight loss with exercise, overweight youth often did not view increasing exercise as a reasonable treatment or prevention strategy. One boy said “*if you run like a mile it's really going to not seem like you would lose anything—I mean like it's really not going to change your life” (7th/8th grade, overweight, male).* In contrast, normal weight participants offered a different perspective in making statements such as, “*it is about committing to staying healthy and always being active. Because it's not really easy because you cannot just be active once, you have to be doing it every single day” (7th/8th grade, normal weight, female)*. Beyond the realization that staying healthy was a lifelong commitment, normal weight youth more often voiced the need for outside support, including having counselors and family members with whom they can talk. 

One exception to the contrasting perspectives on long-term weight management when comparing overweight and normal weight participants were overweight youth who had witnessed the weight loss success of a close friend or family member. Youth who were familiar with someone who lost a substantial amount of weight and became healthy expressed views more similar to the normal weight children, in recognizing that weight loss was a slow and highly controlled process and not a quick fix solution. One overweight male commented “*My dad's like inspiring because he used to be like really fat and then he lost 150 pounds and now he's healthy*.” When other participants in the conversation who were overweight asked whether the weight loss was shocking or sudden *(“was it just like whoa?”),* he went on to describe that *“he gave up sweets for Lent and then a couple of weeks later he decided that it was really helping us so he gave up sweets for good.”*

When discussing the consequences of being overweight or obese, overweight participants focused on current life events, such as not being able to fit on amusement park rides or holding back the team in gym class, rather than serious health conditions that might arise in the future. This was in contrast to normal weight participants who voiced a connection between lifestyle and quality of life. One normal weight male conveyed this clearly when he stated *“if you do not eat healthy and exercise you will die sooner than if you do eat healthy and exercise. If you eat healthy and exercise, you'll live a better life.”*

 Although limited understanding of lifestyle and weight change in the short term was demonstrated across focus group discussions, many overweight youth did not express familiarity with the long-term consequences of poor diet and being overweight, nor did they view their lifelong health as a concern. Particularly among the 4th/5th grade children who were overweight, most stated that their health was “fine” or “good,” and several participants also indicated that overweight youth would naturally become skinny without lifestyle change. For example, youth frequently referred to friends and family members who were fat and “*all of a sudden*” became “*pure skinny*” or “*pure muscle.*”

## 4. Discussion

We analyzed data from focus group transcripts to explore attitudes, beliefs, and knowledge of youth about healthy lifestyles and how such behaviors are related to the causes and consequences of obesity. Exploring youth's familiarity with obesity and its consequences will allow for more informed development of child targeted, community-wide obesity interventions. Most youth reported that they recognized obesity as a problem, in contrast to a study conducted in 2000, in which youth were indifferent to the topic [14]. However, even if they could recognize what it meant to be “overweight,” youth who were themselves overweight did not consider themselves to be so, suggesting that youth do not see themselves as susceptible to becoming obese. This disconnect is consistent with previous research which found that body weight underestimation is greatest among youth with higher BMI [15, 16].

Youth who were overweight attributed being overweight to external causes such as slow metabolism and genetics, whereas most of the normal weight youth perceived being overweight as resulting from alterable lifestyle factors. This discrepancy in youth's understanding of the causes of obesity between weight status groups has to our knowledge not previously been shown. The belief in an external (and potentially nonmodifiable) locus of control is particularly concerning because this belief has previously been correlated with continued weight gain into adulthood [17]. Increases in youths' nutritional knowledge has often failed to produce behavior change [18], which may be explained by overweight youths' tendencies to attribute their weight to nonmodifiable attributes. 

Difficulty in achieving behavior change among youth could also result from low familiarity with or perceived severity of the long-term health consequences of obesity. Our analyses demonstrate that although youth have knowledge about what healthy and unhealthy diets entail, they express that a healthy lifestyle has low appeal and do not connect their own lifestyle with the development of obesity. Overweight youth in particular often view long-term lifestyle modification as unrealistic and, thus, express frustration and helplessness that decreases their motivation to change their behavior. It is possible that obesity prevention education that includes the benefits of healthy diets, particularly attributes designed to have immediate appeal to youth (e.g., healthier skin, feeling stronger, feeling happier, etc.), would make these education efforts more successful.

There were some limitations of the composition of the focus group discussions that may influence the generalizability of our findings. There was an uneven distribution of weight status and age across groups (e.g., more groups with overweight than normal weight children), and none of the focus group discussions included 4th/5th grade children of normal weight. Additionally, because of the group-oriented setting, participants may have responded in a socially desirable manner, which may have introduced bias into the transcripts. Because the moderator was skilled at working with children in this type of setting, this was unlikely to affect our data. Furthermore, youth provided detailed accounts and challenged each other's opinion, which indicated a sense of openness among the focus group participants and demonstrates the generation of good quality data. 

The results of these focus group discussions display the complex and evolving nature of youth attitudes towards obesity prevention efforts and their understanding of the causes and consequences of obesity. Obesity is now accepted and acknowledged as a problem amongst youth and by youth. However, attitudes towards prevention of obesity through employment of healthier habits remain mixed, with primarily negative connotations around healthy lifestyle choices. 

## 5. Conclusions

We found that most youth in the state of Georgia (USA) recognized obesity as a problem yet failed to connect their behavior with the development of obesity. More overweight youth attributed being overweight to external causes, such as slow metabolism or genetics, rather than alterable lifestyle behaviors, such as diet and physical activity. They also did not tend to see sustained lifestyle change as desirable or achievable. It is well documented that excessive caloric intake and inadequate physical activity are important contributors to weight gain and obesity. Our study suggests that youth may benefit from education designed to increase the desirability of healthy habits, either from gaining benefit or from avoiding consequences, specifically linking lifestyle choices with weight-related outcomes. For overweight youth in particular, successful role models seem to be critical to their beliefs that change to a healthier state is achievable. Future research is needed to evaluate whether increasing youth understanding and awareness in these areas will lead to positive behavior change.

## Figures and Tables

**Table 1:** Distribution of participants (*n* = 41) in the focus groups (*n* = 10) separated by weight status, age, and gender.

	4th and 5th grades (Ages 9–11 years)	7th and 8th grades (Ages 12–14 years)
Weight status: normal		
Male	0	10
Female	0	4
Weight status: overweight		
Male	3	10
Female	6	8

**Table 2:** Example of coding scheme for theme^1^ of food and healthy eating.

(1) Food and healthy eating	
(1.1) Healthy versus unhealthy foods	
(1.1.1) Easily identifies healthy food (1.1.2) Difficulty in identifying healthy food (1.1.3) Easily identifies unhealthy food (1.1.4) Difficulty in identifying unhealthy food (1.1.5) Enjoyment of healthy food (1.1.6) Dislike of healthy food (1.1.7) Enjoyment of healthy food (1.1.8) Dislike of healthy food	
(1.2) Consequences of eating healthy food	
(1.2.1) Weight (1.2.2) Risk of chronic disease (1.2.3) Intellectual/school performance (1.2.4) Other health benefits and risks (i.e., bone health)	

^1^Food and healthy eating was the way that the theme was written in the codebook.
